# FoxA and LIPG endothelial lipase control the uptake of extracellular lipids for breast cancer growth

**DOI:** 10.1038/ncomms11199

**Published:** 2016-04-05

**Authors:** Felipe Slebe, Federico Rojo, Maria Vinaixa, Mar García-Rocha, Giorgia Testoni, Marc Guiu, Evarist Planet, Sara Samino, Enrique J. Arenas, Antoni Beltran, Ana Rovira, Ana Lluch, Xavier Salvatella, Oscar Yanes, Joan Albanell, Joan J. Guinovart, Roger R. Gomis

**Affiliations:** 1 Institute for Research in Biomedicine (IRB Barcelona), The Barcelona Institute of Science and Technology, Barcelona 08028, Spain; 2 Cancer Research Programme, IMIM (Hospital del Mar Medical Research Institute), Barcelona 08003 Spain; 3 Pathology Department, IIS-Fundación Jimenez Diaz, Madrid 28040, Spain; 4 Centre for Omic Sciences, Universitat Rovira i Virgili, Reus 43204, Spain; 5 Department of Electronic Engineering, Universitat Rovira i Virgili, Tarragona 43003, Spain; 6 Spanish Biomedical Research Center in Diabetes and Associated Metabolic Disorders (CIBERDEM), Madrid 28029, Spain; 7 Medical Oncology Service, Hospital del Mar, Barcelona 08003, Spain; 8 Medical Oncology Service, Hospital Clinico, Valencia 46010, Spain; 9 Institució Catalana de Recerca i Estudis Avançats (ICREA), Barcelona 08010, Spain; 10 Universitat Pompeu Fabra, Barcelona 08003, Spain; 11 Department of Biochemistry and Molecular Biology, Universitat de Barcelona, Barcelona 08028, Spain

## Abstract

The mechanisms that allow breast cancer (BCa) cells to metabolically sustain rapid growth are poorly understood. Here we report that BCa cells are dependent on a mechanism to supply precursors for intracellular lipid production derived from extracellular sources and that the endothelial lipase (LIPG) fulfils this function. LIPG expression allows the import of lipid precursors, thereby contributing to BCa proliferation. LIPG stands out as an essential component of the lipid metabolic adaptations that BCa cells, and not normal tissue, must undergo to support high proliferation rates. LIPG is ubiquitously and highly expressed under the control of FoxA1 or FoxA2 in all BCa subtypes. The downregulation of either LIPG or FoxA in transformed cells results in decreased proliferation and impaired synthesis of intracellular lipids.

## Abstract (web summary)

Deregulation of lipid metabolism in cancer cells is critical to the maintenance of certain malignant features. Here, the authors show that the proliferation of breast cancer cells depends upon the extracellular activity of the endothelial lipase enzyme LIPG whose expression is regulated by the FoxA family of transcription factors.

## Graphical abstract

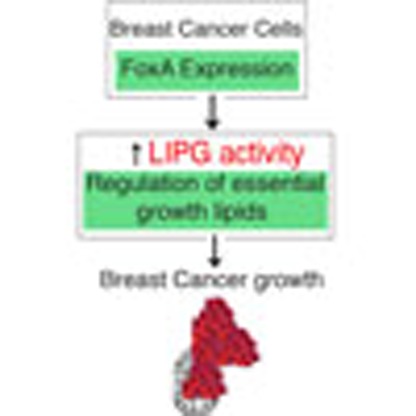

Cancer cells undergo metabolic reprogramming in order to produce biosynthetic precursors, such as ATP, lipids, nucleotides and amino acids, which are required to sustain the energy and substrate demands for rapid growth and proliferation^1^,^2^. Among the various metabolic deregulations in cancer, lipid metabolism emerges as a critical pathway for the maintenance of cell survival, growth and migration^3^. Thus research devoted to understanding the mechanisms of lipid metabolism adaptation in breast cancer (BCa) is highly relevant if we are to devise novel strategies to improve the control of this disease.

The FoxA protein sub-family belongs to the forkhead box (Fox) transcription factor family and comprises FoxA1, FoxA2 and FoxA3. FoxA transcription factors are critical regulators of tissue development, tissue function and metabolism^4^. In the mammary gland, FoxA1 contributes to the differentiation of luminal epithelial cells and co-regulates the hormonal response to estrogen and androgen^5^,^6^,^7^,^8^,^9^. In liver and pancreas, FoxA transcription factors are key controllers of metabolism^8^,^9^. Given the role of FoxA1 in differentiation of luminal epithelial cells and its transcriptional control of metabolic genes and pathways, we study the potential function of FoxA family of transcription factors in BCa metabolism.

Here we reveal that FoxA1 and the transcription factors family regulate the expression of the endothelial lipase enzyme (LIPG), a member of the lipoprotein lipase family. LIPG supports BCa cell lipid addiction and loss of its activity impairs tumour growth.

## Results

### FoxA1 and FoxA2 in BCa growth

**Figure 1:**
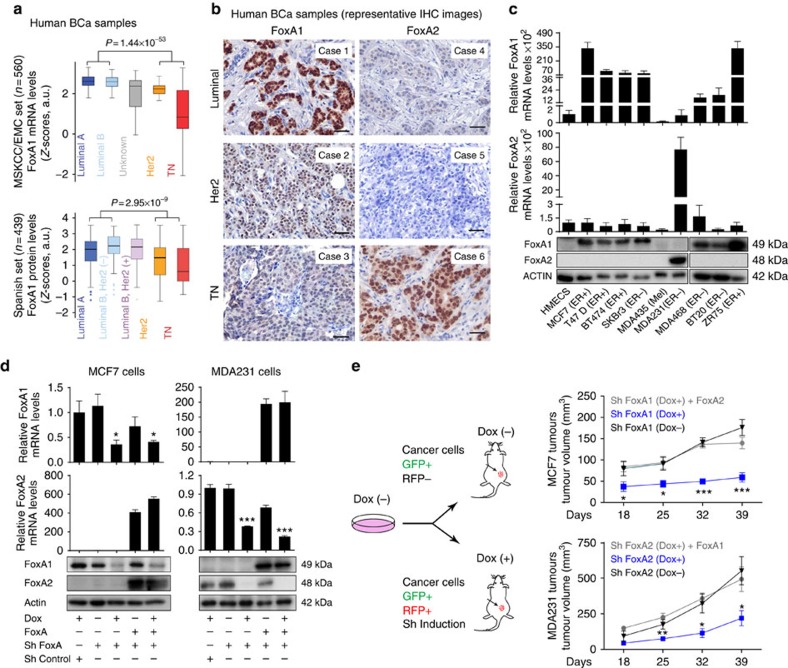
FoxA1 and FoxA2 in BCa growth. (**a**, top) FoxA1 mRNA expression in the MSKCC/EMC set. BCa samples were stratified in Luminal A, Luminal B, Her2, triple negative and unknown subgroups. The unknown group represents specimens that were not classified in any group. (bottom) FoxA1 protein levels by IHC staining in Luminal, Her2 and triple negative samples in the Spanish BCa set (cohort of 439 BCa patients). Data is average±s.d. (**b**) FoxA1 and FoxA2 IHC staining in FFPE human specimens representative of the different BCa subtypes. Six independent cases are depicted. FoxA1 and FoxA2 are expressed mainly in the nuclei of tumour cells. Scale bar, 50 μm. (**c**) FoxA1 and FoxA2 mRNA expression analysis by qRT-PCR and protein expression by western blot in human BCa cell lines compared with HMECs. *T*-test was used. Data are average±s.e.m.; *n*= 3. Of note, MDA435 are of melanoma origin. (**d**) FoxA1 and FoxA2 expression in MCF7, MDA231 and their derivatives cells by qRT-PCR and western blot. FoxA1 and FoxA2 depletion was achieved with a doxycycline-inducible short hairpin vector. FoxA-depleted cells were rescued by expression of FoxA2 in MCF7 cells or FoxA1 in MDA231 cells. Cell populations were cultured in the presence or absence of doxycycline for 6 days. *P* value is the result of *T*-test. Data are average±s.e.m.; *n*=3. *P≤0.05, ***P≤0.001 (**e**, left) Schematic representation of MDA231 and MCF7 cells grown without doxycycline and inoculated in Balb/c nude mice treated with or without doxycycline to induce the expression of the indicated FoxA short hairpins. All tumour cell lines have GFP constitutive expression, and tRFP concomitantly with the short hairpin were expressed in doxycycline treated tumours. (right) Tumour growth of the indicated cell populations inoculated in Balb/c nude mice are determined at the indicated time points. *P* value is the result of *T*-test. Data are average±s.e.m.; *n*= 5–8 tumours. *P≤0.05, **P≤0.01, ***P≤0.001. FFPE, formalin-fixed paraffin-embedded.

The importance of FoxA1 in BCa cells differentiation and its contribution to controlling the expression of metabolic genes in several other tissues makes this transcription factor a highly attractive target to explain the metabolic alterations reported in BCa. For these reason, we decided to ascertain the metabolic processes controlled by FoxA1 in BCa. We first confirmed the association between high FoxA1 expression (mRNA and protein) and luminal subtype (Fig. 1a). To this end, we used two cohorts of primary breast tumours with annotated clinical features and follow-up. The MSKCC/EMC BCa data set is based on gene expression profiles from an original series of 560 cases^10^, whereas the Spanish BCa data set (*n*=439) is a tissue microarray of formalin-fixed paraffin-embedded stage I–III breast tumour specimens^11^ (details provided in Methods Section). High *FoxA1* gene expression significantly correlated with high expression of well-established luminal markers, such as *GATA3* and *ESR1*, in primary tumours (Supplementary Fig. 1a). Next we explored FoxA1 expression beyond the luminal subtype. Lower FoxA1 expression was observed in non-luminal tumours (Fig. 1a,b); however, a subset also expressed higher FoxA1 levels (Supplementary Fig. 1b and Supplementary Table 1). Given that FoxA2, in conjunction with FoxA1, is also involved in the regulation of several metabolic pathways, we determined the expression of this factor in BCa samples. Unfortunately, no FoxA2 probes in the Affymetrix platform used in the MSKCC/EMC data set provided a reliable interpretation. To overcome this limitation, we used tissue arrays of early BCa samples (Spanish BCa set). Histological examination of FoxA2-stained tissue microarray slides from the Spanish BCa set revealed the expression of this factor in six non-luminal samples, which were scored as FoxA1**−** (examples in Fig. 1b and summarized in Supplementary Table 1). Collectively, the number of FoxA+ BCa samples detected by immunohistochemistry accounted for 81.3% of all samples in the Spanish BCa set (Supplementary Table 1), which represent a significant proportion of BCa and point to the participation of FoxA in this disease, beyond to its involvement in differentiation and control of hormonal responses.

Next, we extended our analysis to BCa cell lines for further mechanistic studies. We compared FoxA1 and FoxA2 mRNA expression in four estrogen receptor positive (ER+) (MCF7, T47D, BT474 and ZR75) and four estrogen receptor negative (ER−) (SKBR3, MDA468, BT20 and MDA231) BCa cell lines, a cell line of melanoma origin (MDA435), and human mammary epithelial cells (HMECs). Of note, two of the BCa lines tested were HER2+ (BT474 and SKBR3) (Fig. 1c). All ER+ BCa cells (MCF7, T47D, BT474 and ZR75), the ER−/HER2+ SKBR3 and both triple negative-like MDA468 and BT20 cell lines expressed FoxA1. Interestingly, MDA231 triple negative-like cells expressed high levels of FoxA2 but not FoxA1, and the non-tumour HMECs did not express these factors (Fig. 1c). No BCa cells co-expressed these two proteins (Fig. 1c). Our results suggest that the expression of FoxA transcription factors is a common feature of breast tumours, as well as of BCa cell lines. This notion implies that FoxA factors play a major role in BCa growth, independently of luminal fate specification.

To examine the molecular basis of the contribution of FoxA1 and FoxA2 to BCa growth, we engineered constitutive GFP-luciferase-expressing MCF7 and MDA231 cells with a doxycycline-inducible short-hairpin RNA (sh-RNA) vector targeting either FoxA1 or FoxA2. Doxycycline addition to the cell culture media decreased FoxA expression in both cell lines compared with control cells (ShControl (Dox+) and Sh FoxA1 or Sh FoxA2 (Dox−))(Fig. 1d), with the concomitant expression of tRFP (Supplementary Fig. 1c). Of note, there was no gain of expression of FoxA2 in FoxA1-depleted cells or vice versa (Fig. 1d). Interestingly, cancer cell proliferation was impaired *in vitro* upon depletion of either FoxA1 or FoxA2 in MCF7 and MDA231 cells, respectively (Supplementary Fig. 1d,e). Similarly, when Balb/c nude mice implanted with xenograft tumours from the above described cellular populations were treated with doxycycline and the short hairpins were induced, striking differences in tumour growth were observed. FoxA1-depleted MCF7 and FoxA2-depleted MDA231 tumour growth was blunted (Fig. 1e and additional controls in Supplementary Fig. 1f. Experimental details in the Supplementary Methods Section). Collectively, these observations confirm that FoxA1 or FoxA2 expression is required for BCa growth.

Previous studies indicate that FoxA1 and FoxA2 transcriptionally regulate common genes in the liver and pancreas that are central to development and metabolism. We therefore hypothesized that crossed expression of FoxA factors could rescue tumour growth by restoring the expression of essential metabolic genes. To this end, we engineered doxycycline-driven shFoxA1 MCF7 cells to express exogenous FoxA2 and doxycycline-driven shFoxA2 MDA231 cells to express exogenous FoxA1 (Fig. 1d). Interestingly, when these BCa modified cells were implanted in Balb/c nude mice and FoxA depletion was induced with doxycycline, the sustained expression of another FoxA factor (FoxA2 in MCF7 and FoxA1 in MDA231 cells) was sufficient for tumours to continuously grow (Fig. 1e and additional controls in Supplementary Fig. 1f). Quantitative real-time PCR (qRT-PCR) analysis confirmed FoxA expression in the distinct tumour populations *ex-vivo* (Supplementary Fig. 1g). These results showed that retention of minimal levels of FoxA1 or FoxA2 expression is necessary for BCa cell growth.

### FoxA1- and FoxA2-regulated transcripts for BCa growth

**Figure 2:**
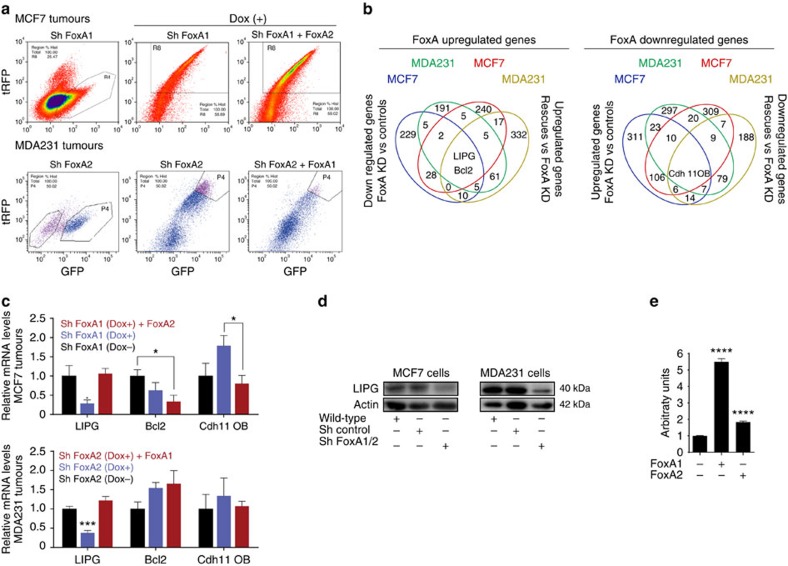
A genomic approach to identify FoxA1- and FoxA2-regulated transcripts in MCF7 and MDA231 cells. (**a**) FACS profiling of MCF7 and MDA231 cells derived from tumours isolated from mice on the basis of the expression of GFP+ and RFP− (control group) or GFP+ and tRFP+ (knockdown and rescue groups). (**b**) Representation of the transcripts up- and downregulated by FoxA in MCF7 and MDA231 cells isolated from tumours. Up- and downregulated transcripts present a Bayesian false discovery rate below 5% and fold change >2.5. (**c**) LIPG, Bcl2 and Cdh11OB mRNA levels of the indicated genetically modified MCF7 and MDA231 tumour xenografts analysed by qRT-PCR. *P* value is the result of *T*-test. Data are average±s.e.m.; *n*= 5–8 tumours. *P≤0.05, ***P≤0.001. (**d**) LIPG protein expression in constitutive shFoxA1 MCF7 or shFoxA2 MDA231 cells. (**e**) Promoter reporter assay in HEK 293 cells. Cells were transfected with LIPG promoter reporter and FoxA1 or FoxA2 expressing vectors when indicated. *P* value is the result of *T*-test. Data are average±s.e.m.; *n*=3. ****P≤0.0001.

Next, we focused on the identification of genes under the control of FoxA1 and FoxA2 with the capacity to support tumour growth in xenograft tumours from implanted MCF7 and MDA231 cells, with a specific focus on metabolic functions. To this end, we isolated cells derived from non-depleted (Dox−) FoxA1 or FoxA2 MCF7 and MDA231 tumours (control groups, GFP+/tRFP−), FoxA1- and FoxA2-depleted MCF7 and MDA231 tumours (GFP+/tRFP+), and the corresponding FoxA family-rescued tumours (GFP+/tRFP+), respectively (Fig. 2a). Tumours surgically removed from the mouse were disaggregated and GFP (cancer cell-specific), and tRFP expression (FoxA1- and FoxA2-depleted cell-specific) was used to isolate human cancer cells from mouse tumour stroma cells (Fig. 2a) in order to purify RNA for subsequent transcriptomic analysis (GSE61164 and Transcriptomic Analysis in Methods Section). Among the genes under the control of FoxA1 and FoxA2 in the six cellular populations, we identified LIPG and BCL2 as positively regulated while CDH11 was negatively regulated (summarized in Fig. 2b; details in Supplementary Table 2 and methods). We applied stringent criteria to our analyses to prevent false positive hits (2.5-fold change and false discovery rate <0.05). BCL2 expression is associated with anti-apoptotic functions in cancer^12^, whereas cadherin CDH11 induces apoptosis^13^. LIPG is an endothelial lipase with phospholipase activity. It is involved in lipoprotein metabolism, and its levels have been associated with testis cancer^14^,^15^,^16^. However, to date, LIPG has not been linked to tumour growth. Robust changes in the mRNA expression of LIPG, but not BCL2 or CDH11, were confirmed by qRT-PCR (Fig. 2c). Next, we confirmed that FoxA factors regulate LIPG and its promoter activity (Fig. 2c–e).

### LIPG expression in BCa

**Figure 3:**
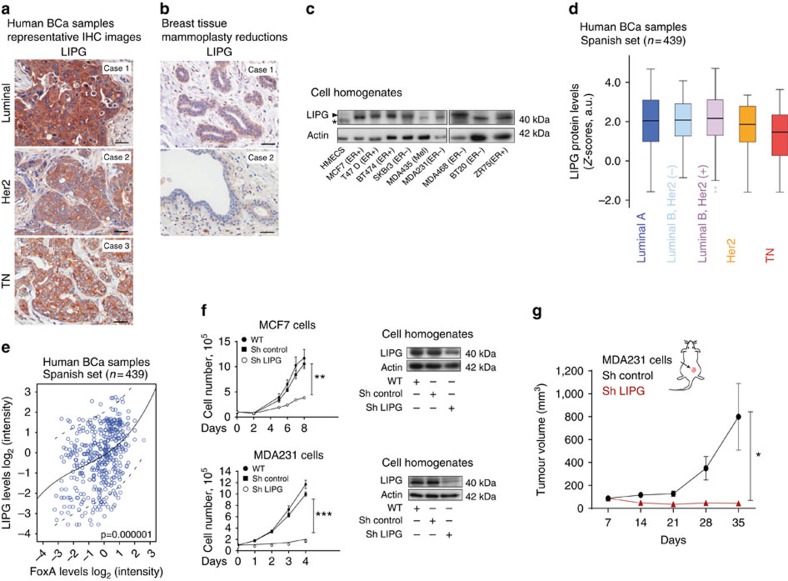
LIPG contributes to BCa growth. (**a**) Representative LIPG IHC staining on primary BCa tissues (cohort of 439 BCa patients). LIPG is expressed in the cytoplasm of tumour cells. Faint staining is also detected in the extracellular area. Scale bar, 50 μm. (**b**) Representative LIPG IHC staining in normal breast tissue from mammoplasty reductions. Weak LIPG expression occurs in epithelial cells from ducts and lobuli. Scale bar, 50 μm. (**c**) LIPG protein expression in human cancer cell lines compared with HMECs. Actin was used as loading control. *Unspecific band. Of note, MDA435 are of melanoma origin. (**d**) LIPG protein levels by IHC staining in Luminal, Her2, and triple negative samples in the Spanish BCa set (cohort of 439 BCa patients). Data is average±s.d. (**e**) Spearman correlation (*P*=0.000001) between FoxA and LIPG IHC staining intensities in Spanish BCa set (cohort of 439 BCa patients). (**f**) Left panel, *in vitro* proliferation curves of MCF7 and MDA231 cells transduced with a control or a LIPG short hairpin. Data are average±s.e.m.; *n*=3. (right) LIPG protein expression in shLIPG MCF7 and shLIPG MDA231 cells. The blot shown is representative of three independent experiments. *P* value is the result of *T*-test.**P≤0.01, ***P≤0.001. (**g**) Tumour growth of the indicated cell populations inoculated in Balb/c nude mice are determined at the indicated time points. *P* value is the result of *T*-test. Data are average±s.e.m.; *n*= 6–8 tumours. *P≤0.05.

Next, we showed that LIPG expression in primary tumours was specific to BCa tumour cells and not to other stroma cellular entities (Fig. 3a). Subsequently, we tested LIPG expression in normal breast epithelia and interrogated 20 samples from mammoplasty reductions. Normal breast epithelial cells showed a lower expression of LIPG than cells from tumour specimens (Fig. 3b). Similar results were obtained for LIPG protein levels in a panel from BCa lines compared with HMEC cells. Of the cellular populations tested, the eight BCa cell lines expressing FoxA1 or FoxA2 had very high levels of LIPG protein compared with the melanoma MDA435 cell line and the human epithelial cell (Fig. 3c). Consistent with this observation, 83.8% of BCa samples in the Spanish tumour cohort were LIPG+ (Fig. 3d and Supplementary Table 3), and LIPG expression correlated with FoxA expression (Spearman correlation; *r*=0.477, *P*=0.000001; Fig. 3e). Further analysis showed that LIPG expression levels in primary tumours do not have the capacity to stratify patients for differential risk of overall or disease-free survival (Supplementary Fig. 2a) and are not dependent on estrogen signalling (Supplementary Fig. 2b), thus reinforcing the notion that LIPG is essential for BCa growth.

LIPG is a phospholipase located in the cytosol and cellular membrane and has been shown to hydrolyse extracellular phospholipids from high-density lipoprotein that are afterwards incorporated into intracellular lipid species thus providing lipid precursors of cell metabolism^17^,^18^. Thus we questioned whether LIPG regulates essential lipid intake in BCa and whether it is necessary for proliferation. To validate this hypothesis, we genetically downregulated the expression of this protein in MCF7 and MDA231 cells by means of sh-RNA (Fig. 3f and Supplementary Fig. 2c). LIPG depletion blunted BCa cell capacity to proliferate *in vitro* (Fig. 3f), as previously observed in FoxA-depleted cells (Supplementary Fig. 1d,e), and caused a reduction in invasion and self-renewal properties (Supplementary Fig. 3a–d). Similarly, LIPG-depleted cells were unable to grow tumours *in vivo* (Fig. 3g).

### LIPG induces BCa cells lipid metabolic reprograming

**Figure 4:**
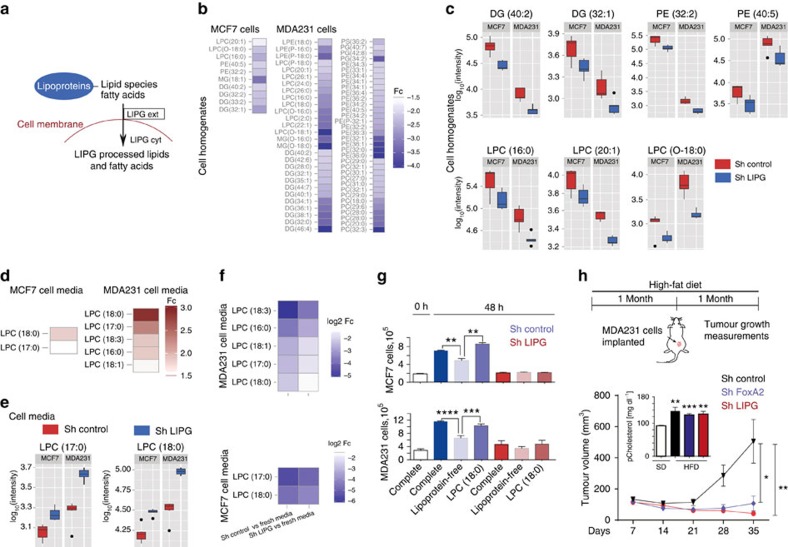
LIPG regulates the uptake of lipids in BCa cells inducing a lipid metabolic reprograming. (**a**) Schematic representation of LIPG action. (**b**) Heat map representation of the downregulated (blue) lipids identified by MS/MS in the cell homogenates of MCF7 or MDA231 LIPG-depleted cells compared with shControl cells. Depicted lipids have a fold change >1.5 and *P* value<0.05 using the Welch's *t*-test *n*=5. (**c**) Downregulated lipid species (previously identified in **b**) that are common to LIPG-depleted MCF7 and LIPG-depleted MDA231 cells. ShControl cells (red box), and shLIPG (blue box). *P* values are <0.05 and calculated using Welch's *t*-test, *n*=5. Whiskers extend to a maximum of 1.5 × IQR beyond the box. (**d**) Heat map representation of the upregulated (red) lipids identified by MS/MS in the media of MCF7 or MDA231 LIPG-depleted cells compared with the corresponding shControl cells. Characterized lipids have a fold change >1.5 and *P* value<0.05 using the Welch's *t*-test *n*=5. (**e**) Upregulated lipid species in the media (previously identified in **d**) that are common to LIPG-depleted MCF7 and LIPG-depleted MDA231 cells. ShControl cells (red box), and shLIPG cells (blue box). *P* values are <0.05 and calculated using Welch's *t*-test, *n*=5. Whiskers extend to a maximum of 1.5 × IQR beyond the box. (**f**) Heat map representation of the MS/MS downregulated (blue) lipids in the cell media of MCF7/MDA231 LIPG-depleted or shControl cells (as described in **d**) compared with fresh medium (without cell incubation). Depicted lipid species have a log_2_ fold change>1.5 and *P* value<0.05 using the Welch's *t*-test *n*=5. (**g**) MDA231 and MCF7 cell growth for 48 h in complete medium: medium containing 10% FBS 10%); lipoprotein-free medium: medium containing 10% free lipoprotein FBS; and LPC (18:0): medium containing 10% free lipoprotein FBS and 20 μM of LPC (18:0). *P* value is the result of *T*-test. Data are average±s.e.m.; *n*=3. **P≤0.01, ***P≤0.001, ****P≤0.0001. (**h**) Above, schematic representation of the experimental protocol used. (bottom) Tumour growth of the indicated cell populations inoculated in Balb/c nude mice treated with high-fat diet (HFD) are determined at the indicated time points. *P* value is the result of *T*-test. Data are average±s.e.m.; *n*= 6–8 tumours. *P≤0.05, **P≤0.01. Inside graph, plasma cholesterol levels of animals treated with standard diet (SD) or HFD. *P* value is the result of *T*-test. Data are average±s.e.m.; *n*= 4 animals per group. **P≤0.01, ***P≤0.001.

Next, we sought to identify whether the lack of LIPG caused a cellular metabolic reprograming in the above selected BCa cell models (MCF7 and MDA231 cells) (Fig. 4a). We used a liquid chromatography-mass spectrometry-based untargeted lipidomic analysis to compare LIPG-depleted versus control BCa cell homogenates. This approach allowed us to quantify >7,000 metabolic features (defined as molecular entities with a unique mass/charge and retention time) in positive and negative ionization modes. On the basis of accurate mass information, altered metabolic features (*P* value <0.05) in LIPG-depleted cells accounted for 21% and 8% of the lipid landscape in MDA231 and MCF7 cells, respectively. LIPG depletion induced a significant reduction in lipid species both in MCF7 and MDA231 cells (that is, 85 and 96% of the altered species, respectively), the latter involving a greater number of lipids (Supplementary Fig. 4a). Supportingly, a reverse profile is observed when LIPG gain of function is evaluated (Supplementary Fig. 4b). Downregulated lipid families in LIPG-depleted cells included mainly glycerophospholipids such as phosphatyl ethanolamines (PEs) and phosphatyl cholines (PCs), and glycerolipids such as triacylglycerols (TGs) and diacylglycerols (DGs), as shown by accurate mass information (Supplementary Fig. 4d). We next structurally identified a large proportion of these lipids by means of MS/MS experiments (Fig. 4b), which confirmed the depletion of PCs, LPCs, PEs, LPEs, PGs, DGs and MGs in MCF7 and MDA231 cells. In particular, a specific LIPG lipid signature shared by the two cell lines was found to include the following: DG (40:2); DG (32:1); PE (32:2); PE (40:5); LPC (O-18:0); LPC (16:0); and LPC (20:1) (Fig. 4c). Some of these lipid families are pivotal in cancer malignancy^19^,^20^. Collectively, these results suggest that LIPG shapes lipid metabolism in BCa cells to support cellular growth requirements. Interestingly, FoxA depletion in both MCF7 and MDA231 cells led to a lipid metabolic reprograming similar to LIPG depletion (Supplementary Fig. 4c–e).

LIPG location has been shown to be functional on the outer face of the cellular membrane (Fig. 4a)^18^, thus we postulated the possibility that BCa cells are dependent on LIPG function to access extracellular lipids to support their growth needs. To test this notion, we profiled the media of control and LIPG-depleted MCF7 and MDA231 cells following the same liquid chromatography-mass spectrometry-based untargeted lipidomic approach as for cell homogenates. LIPG depletion prevented the absorption of particular lipids from the media (Supplementary Fig. 4a). The structural identification of the lipids by MS/MS confirms the absence of degradation of glycerophospholipids belonging to the LPC class in both MCF7 and MDA231 cells, which is depicted by higher levels in the media of these species in LIPG-depleted when compared with control cells (Fig. 4d,e). Interestingly when we analysed the LPCs species in the media of control and LIPG-depleted cells and compared with fresh media (without cells), all LPC species from control cell media were decreased. This reduction was weaker in the media of Sh LIPG cells, indicating that LIPG-depleted cells have a defect in processing and importing of pre-existing lipid species from the medium (Fig. 4f).

Finally, we evaluated which of the commonly identified potential substrates of LIPG sustains BCa cell proliferation. Initially, we confirmed that the growth of MCF7 and MDA231 cells is impaired when grown *in vitro* in lipoprotein-depleted media (Fig. 4g). Next we tested the capacity of LPC (18:0) to rescue BCa cell growth in the absence of lipoproteins and confirmed that this lysophosphatidylcholine was able to restore the cells' capacity to proliferate (Fig. 4g). In accordance, this process was dependent on LIPG expression (Fig. 4g). Similarly, LIPG-depleted cells were not able to grow *in vivo* in animals fed with high-fat diet (Fig. 4h) indicating that LIPG is indispensable to process the extracellular lipids and mediate their uptake by the cells, irrespectively of the concentration of lipid substrates in circulation, a phenotype also observed in FoxA-depleted cells (Fig. 4h).

### LIPG activity supports BCa growth

**Figure 5:**
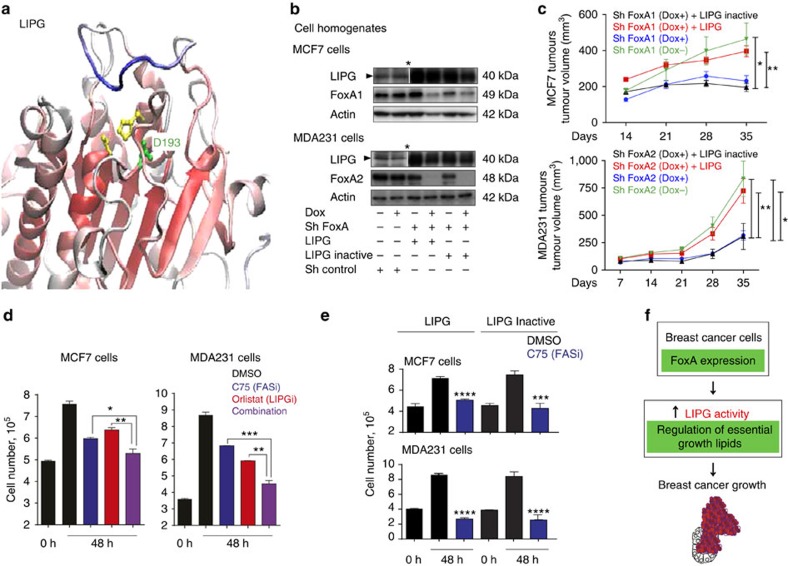
LIPG activity is essential for BCa growth. (**a**, top) Homology 3D structural model of LIPG (backbone coloured according to the QMEANlocal parameter values; red residues with low error). The heavy atoms of the three catalytic residues are shown explicitly and the residue mutated in this study is shown in green (Asp 193). (**b**) FoxA1, FoxA2 and LIPG protein expression in MCF7, MDA231 and their derivative cells determine by western blot. FoxA1 and FoxA2 depletion was achieved with a doxycycline-inducible short hairpin vector. FoxA-depleted cells were rescued by expression of a WT or Inactive LIPG. Cell populations were cultured in the presence or absence of doxycycline for 6 days. *blots represent different exposition times. (**c**) Tumour growth of the indicated cell populations inoculated in Balb/c nude mice are determined at the indicated time points. *P* value is the result of *T*-test. Data are average±s.e.m.; *n*=5–8 tumours. *P≤0.05, **P≤0.01. (**d**) MDA231 and MCF7 cell growth for 48 h treated with DMSO (control), FAS inhibitor (C75) and/or lipase inhibitor (Orlistat). For MDA231 cells C75 was used at a final concentration of 10 μg ml^−1^ and for MCF7 cells 8 μg ml^−1^. Orlistat was used at a final concentration of 30 or 10 μg ml^−1^ in MCF7 or MD231 respectively. *P* value is the result of *T*-test. Data are average±s.e.m.; *n*=3.*P≤0.05, **P≤0.01, ***P≤0.001. (**e**) Forty-eight hours cell growth of MDA231 or MCF7 cells overexpressing exogenous WT or Inactive LIPG. Cells were treated with DMSO (control) and FAS inhibitor (C75) at a final concentration of 20 μg ml^−1^. *P* value is the result of *T*-test. Data are average±s.e.m.; *n*=3.***P≤0.001, ****P≤0.0001 (**f**) Schematic representation showing how FoxA controls LIPG and lipid metabolism to support tumour growth.

The control of LIPG expression by FoxA family transcription factors is likely to increase the availability of lipids required for BCa cell membrane synthesis and signalling cascades, thus facilitating the proliferation and growth of these cells, as well as other traits associated with tumour progression and metastasis. To confirm the central role of LIPG activity in BCa tumour growth, and given the overlapping phenotypes of cell growth impairment, we tested the capacity of LIPG and its catalytic activity to rescue FoxA depletion in tumour growth. To this end, we modelled the three–dimensional (3D) structure of LIPG based on the structure of other family members and confirmed the presence of Asp193, key residue from the catalytic domain of the human enzyme (Fig. 5a). Indeed, the sole mutation of aspartic acid 193 to an asparagine was sufficient to inactivate LIPG^21^. Next, we genetically engineered MCF7 and MDA231 cells containing the doxycycline-driven FoxA1 or FoxA2 sh-RNA expression system and transduced them with either a catalytically active or inactive LIPG expression vector (Fig. 5b). Then, 1 × 10^6^ cells were injected into Balb/c nude mice treated with or without doxycycline (Fig. 5c). Remarkably, the effect of FoxA1 or FoxA2 depletion on tumour growth was rescued only in tumours expressing exogenous wild-type (WT) LIPG but not in those with a catalytically inactive form of the protein (Fig. 5c and additional controls in Supplementary Fig. 5a). Furthermore, gene expression analysis confirmed FoxA depletion and LIPG expression in the tumour populations at the end point of the tumour growth experiment and the rescue was also confirmed in cell culture (Supplementary Fig. 5b,c). Overall, these results suggest that BCa growth requires exogenous lipid precursors and that these are provided, in part, by LIPG activity.

As previous reports showed that *de novo* lipid metabolism is necessary for BCa growth^3^,^22^, we next questioned whether this lipid synthesis was sufficient or, instead, whether exogenous sources are also required to support BCa cell growth and proliferation, as suggested by our experimental data. To this end, we inhibited the activity of fatty acid synthase (FAS) in BCa cells by means of the chemical inhibitor C75 (ref. 23). FAS activity is crucial for *de novo* lipid synthesis in cancer cells^3^,^22^. To test the complementarity of both *de novo* and/or exogenous lipid supplies, we used a C75 concentration causing a 50% reduction in BCa cell growth *in vitro* 48 h post incubation (Fig. 5d and Supplementary Fig. 5d). Similarly, we tested the contribution of LIPG inhibition by means of treatment with a lipase inhibitor, Orlistat^21^. A specific dose causing a 50% reduction in the growth of each BCa cell line was further used (Fig. 5d and Supplementary Fig. 5d). Interestingly, concomitant treatment with FAS and LIPG inhibitors caused an additive effect, blunting BCa cell growth (Fig. 5d). Next, we evaluated whether LIPG activity was sufficient to rescue the chemical inhibition of FAS. To this end, we overexpressed WT and inactive LIPG and grew MCF7 and MDA231 cells in the presence or absence of a high dose of C75 (20 mg ml^−1^), which blocks cell growth (Supplementary Fig. 5d). Complete blockade of FAS was not rescued by LIPG (Fig. 5e). Collectively, our results suggest that both exogenous lipid precursors provided by means of LIPG activity and *de nov*o lipid synthesis mediated by FAS are necessary for BCa cell growth.

## Discussion

Here we reveal that FoxA factors provide a central metabolic growth function by specifically regulating LIPG expression, thereby allowing the acquisition of indispensable extracellular lipids for BCa tumour proliferation. FoxA family of transcription factors are expressed in the vast majority of BCa and FoxA1 is expressed across various BCa subtypes. Moreover we show that, in some cases, its absence is associated with the expression of FoxA2. Interestingly, in addition of FoxA1 contribution to luminal commitment^24^,^25^,^26^,^27^ the factor may drive BCa growth by specifically regulating LIPG levels.

The catalytic activity of LIPG generates extracellular lipid precursors that are imported to fulfill the intracellular production of lipid species (Fig. 5f). LIPG downregulation blocks BCa cell growth, thereby indicating that the import of extracellular lipid precursors is important for the proliferation of these cells. This is a striking observation given that it is generally believed that *de novo* fatty acid synthesis is the main driver of tumour growth^22^. Indeed, our experimental data with LIPG-depleted BCa cells revealed a massive decrease of most intracellular glycerolipid intermediates in the synthesis of TG (PC, PE, PG and DG) and their derivatives (LPC and LPE). Accordingly, certain lipid species (LPC) in the media were not decreased in LIPG-depleted cells as much as in control cells, thus indicating that extracellular lipids are the substrates for intracellular lipid production. In particular, we demonstrate the relevance of extracellular LPC (18:0) for BCa cell proliferation in a lipoprotein-depleted medium, a process dependent on LIPG. In this context, a high-fat diet was shown to rescue the absence of a critical intracellular lipase, Monoacylglycerol lipase, for cancer pathogenesis given cancer cells ability to uptake lipids from the extracellular compartment was functional^19^. Herein, we showed that this rescue mechanism is not functional in BCa cells in the absence of FoxA2 or LIPG. In support of this notion, it is worth noting that extracellular LIPG activity releases fatty acids from high-density lipoprotein phospholipids and these acids are further employed for intracellular lipid production in the human hepatic cell line HepG2 (refs 28, 29).

In conclusion, BCa cells are dependent on a mechanism to supply precursors derived from extracellular sources for intracellular lipid production, and LIPG fulfills this function. Therefore, LIPG stands out as an important component of the lipid metabolic adaptations that BCa cells, and not normal tissue, must undergo to support high proliferation rates. Our results also suggest that *de novo* lipid synthesis is necessary but not sufficient to support lipid production for BCa tumour growth. Accordingly, recent clinical studies demonstrate the association between lipids and lipoproteins in circulation and risk of BCa in women with extensive mammographic density. This observation implies that interventions aimed to reduce them may have effect on BCa risk^30^. All together, these observations make LIPG activity an Achilles heel of luminal and, more importantly, of triple negative/basal-like breast tumours, for which limited therapeutic options are currently available.

## Methods

### Breast cancer cell lines

HMECs were purchased from Lonza and MDA-MB-468, ZR75, MDA-MB-435, MDA-MB-231, MCF7, T47D, BT20 and BT474 cells from ATCC. MDA-MB-435 was used to test BCa cell lines specificity as it is reported to be a melanoma. All cells, except HMECs, were maintained in DMEM supplemented with 10% FBS, 100 mg ml^−1^ streptomycin, 100 U ml^−1^ penicillin and 2 mM L-glutamine. HMECs were maintained in mammary epithelial growth medium (Lonza). Cells were tested for mycoplasma monthly as per laboratory routine.

### Western blot and qRT-PCR analysis

For FoxA1 and FoxA2 detection, cells were lysed with a buffer containing (10% SDS, 60 mM Tris pH 6.8 and 7% DTT). For LIPG detection, cells were lysed using a buffer containing 25 mM Tris-HCl pH 7.4, 25 mM NaCl 1% (v/v), Triton X-100, 0.1% SDS, 10 mM NaF, 10 mM PPi, 1 mM sodium orthovanadate, 0.5 mM EGTA and 20 nM oxadaic acid supplemented with protease and phosphatase inhibitors (Sigma Aldrich). Equal amounts of protein per sample were separated by standard SDS–polyacrylamide gel electrophoresis and transferred to immobilon membranes (Millipore). The membranes were incubated with FoxA1 (Millipore), LIPG (Abcam), FoxA2 (Cell Signaling), ESR1 (Abcam) or actin (Sigma). FoxA1, FoxA2, LIPG and ESR1 antibodies were used at a dilution of 1:1,000 and Actin at a dilution of 1:25,000. To assess changes in expression of selected genes, qRT-PCR was performed using the comparative CT method and an ABI Prism Fast 7900 HT instrument (PE Applied Biosystems). Amplification was performed using TaqMan Gene Expression Assays (Applied Biosystems), following the manufacturer's instructions. FoxA1 (Hs00270129-m1), FoxA2 (Hs00232764-m1), LIPG (Hs00195812-m1) and 18S (Hs99999901-s1) were purchased from Applied Biosystems. All assays were done in triplicate, and 18S mRNA was used as an endogenous control for normalization. Results were analysed using SDS2.3 software (PE Applied Biosystems).

### Overexpression and mutagenesis

Overexpression of FoxA1, FoxA2 and LIPG was achieved by lentivirus. Briefly, the human ORF of each protein was purchased from Open Biosystem, and cDNA was made to obtain a PCR product with recombination arms for insertion in a p-entry clone p221 using the gateway system. FoxA1 primers (Fw: 5′-GGGGACAAGTTTGTACAAAAAAGCAGGCTTCACCATGTTAGGAACTGTGAAG ATGGAAGG-3′; Rev: 5′-GGGGACCACTTTGTACAAGAAAGCTGGGTTCTAGGAAGTGTTTAG GACGGGTCTGG-3′), FoxA2 primers (Fw: 5′-GGGGACAAGTTTGTACAAAAAAGCAGGCTTC ACCATGCTGGGAGCGGTGAAGATGGAAGG-3′; Rev: 5′-GGGGACCACTTTGTACAAGAAAGC TGGGTTTTAAGAGGAGTTCATAATGGGCCGGGA-3′, and LIPG primers (Fw: 5′-GGGGACAAGT TTGTACAAAAAAGCAGGCTTCACCATGAGCAACTCCGTTCCTCTGCTCTG-3′, Rev: 5′-GGGGAC CACTTTGTACAAGAAAGCTGGGTTTCAGGGAAGCTCCACAGTGGGACTGG-3′. After confirmation of cDNA sequence fidelity, the cDNA was transferred into a p-lenti vector using a gateway system. Afterwards, HEK293T cells were seeded and transfected with lentiviral packaging plasmids and the p-lenti FoxA1, FoxA2 or LIPG vectors. The supernatants of the cells were collected, and MCF7 and MDA231 cells were infected and selected with 5 μg ml^−1^ hygromycin to obtain a stable resistant population.

Catalytically inactive LIPG was generated by site-direct mutagenesis using the template p-entry plasmid p221-LIPG, primers (Fw: 5′-CGAATCACAGGTTTGAATCCTGCCGGGCC-3′); (Rev: 5′-GGCCCGGCAGGATTCAAACCTGTGATTCG-3′), and the QuikChange XL Site-directed Mutagenesis Kit (Stratagene). Mutation of aspartic acid 193 to an asparagine was confirmed by DNA sequencing, and the ORF with the mutation was transferred to the p-lenti vector as described before.

### Transcriptomic analysis

Generation of biotinylated complementary RNA (cRNA) probes from RNA samples was achieved following the standard Affymetrix (Santa Clara, CA) GeneChip protocol. cRNA probe was prepared using Total RNA (10 μg) and employing a Custom Superscript Kit (Invitrogen). Isothermal amplification SPIA Biotin System (NuGEN Technologies), for expression profiling, was achieved using 25 ng of RNA per sample. Samples were hybridized with a GeneAtlas Human Genome U-219 array, and Bioconductor was used to generate the statistical analyses for the microarrays^31^. Quantile normalization, background correction, and RMA summarization was achieved following Bioconductor's Affy package instructions. Hybridization was performed at IRB Barcelona's Functional Genomics Facility and statistical analyses at IRB Barcelona's Biostatistics/Bioinformatics Facility.

Comparative genome-wide analysis yielded two hits of genes positively regulated by FoxA1 and FoxA2 (GSE61164). The genes of interest were selected on the basis of the following criteria: (1) decreased expression in MCF7 and MDA231 cells upon FoxA depletion compared with control cells; and (2) increased expression in MCF7 and MDA231 FoxA-rescued cells compared with the respective FoxA-depleted population. In contrast, to classify a gene transcript as negatively regulated by FoxA, we established that a gene probe must meet the following criteria: (1) increased expression in MCF7 and MDA231 cells upon FoxA depletion compared with control cells; and (2) decreased expression in MCF7 and MDA231 FoxA-rescued cells compared with the respective FoxA-depleted population.

All changes were computed when a >2.5 fold change, positive or negative, with a Bayesian false discovery rate below 5% was reached (Supplementary Table 2 and summarized in Fig. 2b). False discovery rates were calculated using a semi-parametric empirical Bayes^32^ procedure based on moderated *t*-tests^33^ as implemented in the limma package of Bioconductor.

### Short hairpin studies in cells and proliferation assays

Briefly, HEK293T cells were seeded and transfected with lentiviral packaging plasmids and TRC1 sh clones for LIPG (5′-CCGGGCCGCAAGAACCGTTGTAATACTCGAGTATTACAACGGTTCTTGCGGCTTTTTG-3′), FoxA1 (5′-CCGGGAACACCTACATGACCATGAACTCGAGTTCATGGTCATGTAGGTGTTCTTTTT-3′) or FoxA2 (5′-CCGGGCCCATTATGAACTCCTCTTACTCGAGTAAGAGGAGTTCATAATGGGCTTTTT-3′), all purchased from Sigma Aldrich. MCF7 and MDA231 cells were infected and selected with 5 μg ml^−1^ puromycin to obtain a stable resistant population that were used for experiments in the following 10 days after selection. After selection, they were recovered with fresh medium, and 10^5^ cells were plated in six-well culture plates. At each time point, cells were trypsinized, collected, and counted using a Scepter 2.0 Cell Counter.

### Breast cancer cell growth in lipoprotein-free medium

5 × 10^5^ MCF7 and MDA231 cells were plated in six-well culture plates and incubated O/N in Dulbeccós modified Eaglés medium (DMEM) supplemented with 10% lipoprotein-free FBS (Gibco), 100 mg ml^−1^ streptomycin, 100 U ml^−1^ penicillin and 2 mM L-glutamine. Then, the medium was replace by: complete medium: DMEM supplemented with 10% FBS (Gibco), 100 mg ml^−1^ streptomycin, 100 U ml^−1^ penicillin and 2 mM L-glutamine; lipoprotein-free medium: DMEM supplemented with 10% lipoprotein-free FBS (Gibco), 100 mg ml^−1^ streptomycin, 100 U ml^−1^ penicillin, and 2 mM L-glutamine, Transferrin (10 μg ml^−1^), Triiodothyronine (0,01 μM) (Sigma), *o*-phosphorylethanolamine (5 μg ml^−1^) (Sigma), glutathione (0,012 μg ml^−1^) (Sigma), hydrocortisone (0,5 μg ml^−1^) (Sigma), EGF (10 ng ml^−1^) (RD), FGF (10 μg ml^−1^) (Gibco), Insulin (20 μg ml^−1^), medroxiprogesterone (10 nM) (Sigma) and BSA-free fatty acids 0,5% (Sigma); LPC (18:0) medium: lipoprotein-free medium supplemented with 20 μM LPC 18:0 (Avanti polar). LPC 18:0 was dissolved in water. The same three media were used in cells depleted of LIPG. After 48 h of incubation cells were trypsinized, collected and counted using a Scepter 2.0 Cell Counter.

### *In vitro* drug assays

A total of 5 × 10^5^ MCF7 and MDA231 cells were plated in six-well culture plates and incubated O/N in DMEM supplemented with 10% lipoprotein-free FBS (Gibco), 100 mg ml^−1^ streptomycin, 100 U ml^−1^ penicillin and 2 mM L-glutamine. Then the media was replace by, complete medium: DMEM supplemented with 10% FBS (Gibco), 100 mg ml^−1^ streptomycin, 100 U ml^−1^ penicillin and 2 mM L-glutamine supplemented with Orlistat (Sigma), (C75 Sigma) or a combination of both drugs. Both drugs were resuspended in DMSO following manufacture indications. Concentrations of drugs are indicated in the figure legends. After 48 h of incubation cells were trypsinized, collected, and counted using a Scepter 2.0 Cell Counter. For tamoxifen treatment cells were plated and incubated in DMEM supplemented with 0,5% FBS (Gibco), 100 mg ml^−1^ streptomycin, 100 U ml^−1^ penicillin and 2 mM L-glutamine supplemented with tamoxifen (6 μM) for 24 h.

### Reporter assays

Renilla and luciferase reporter assays were performed as previously described^34^. The plasmid p-lightSwitch-Promo-Renilla containing the LIPG promoter was used (SwitchGear Genomics, ID:S715324). SFG-TGL Luciferase plasmid (Promega) was included to control for transfection efficiency.

### Inducible sh-RNA systems

The inducible sh-RNA systems were generated as described^35^. First, we selected a hairpin sequence from constitutive short hairpins (TRC1 sh clones Sigma Aldrich, LIPG (5′-CCGGGCCGCAAGAACCGTTGTAATACTCGAGTATTACAACGGTTCTTGCGGCTTTTTG-3′); FoxA1(5′-CCGGGAACACCTACATGACCATGAACTCGAGTTCATGGTCATGTAGGTGTTCTTTTT-3′); FoxA2(5′-CCGGGCCCATTATGAACTCCTCTTACTCGAGTAAGAGGAGTTCATAATGGGCTTTTT-3′) known to work for FoxA1 or FoxA2. We designed and ordered a template oligonucleotide by incorporating miR-30 micro RNA sequences between the sense and the antisense sequence of the selected hairpin and then amplified the fragment to incorporate *Eco*RI and *Xho*l cloning sites. The PCR product was isolated from a PCR gel with ILLUSTRA GFX PCR DNA and the Gel Band Purification Kit (GE Healthcare) and digested with *Eco*RI and *Xho*l at 15–25 °C. The digested product was inserted in a p-Triptz vector, and the ligation reaction was transformed into competent bacteria, which were selected in the presence of ampicillin. Afterwards, HEK293T cells were seeded and transfected with lentiviral packaging plasmids and the generated ptriptz-sh FoxA1- or ptriptz-sh FoxA2-inducible vectors. MCF7 and MDA231 cells were infected with the above-mentioned vectors and selected with 5 μg ml^−1^ puromycin to obtain a stable resistant population.

### Adhesion assay

Cells were labelled with 5 μM of cell Tracker green (Invitrogen), following the manufacturer's instructions, and kept overnight in medium without FBS. Next day, 5 × 10^4^ cells were seeded in triplicates on 10 μg ml^−1^ collagen I- or fibronectin-coated 24-well plates dissolved in DMEM. Two hours (in the case of MDA-MB-231) or 3 hours (in the case of MCF7) after seeding, cells were washed carefully with PBS and fixed with 4% formalin. Cells were visualized and counted using the Olympus ScanR- Xcellence-TIRF widefield fluorescence microscope. Each well was segmented in 11 × 11 squares (in total 121), and one image per square was taken. Images and counting were processed with a custom macro created by Anna Lladó from the Advanced Digital Microscopy Core Facility at IRB Barcelona and designed for the Fiji program. The total number of cells per well is shown. A two-tail Mann–Whitney unpaired *t*-test was used to analyse statistical differences between groups.

### Migration assay

Cells were marked with 5 μM of cell Tracker green (Invitrogen), following the manufacturer's instructions, and kept overnight in medium without FBS. Next day, 5 × 10^4^ cells were seeded in triplicates on fibronectin-coated BD Biocat Cell Culture Inserts (BD Bioscience, cat no 354543) in medium without FBS, while the wells were loaded with complete medium. Before seeding, inserts were hydrated with 250 μl of medium for 30 min at 37 °C. Eight hours after seeding, cells were washed with PBS and fixed with 4% formalin. Cells on the apical side of each insert were scraped off, and migration to the basolateral side was visualized with an Olympus ScanR- Xcellence-TIRF widefield fluorescence microscope. Each insert was segmented in 6 × 6 squares (in total 36), and one image per square was taken. The images shown are the result of the 36 pictures stitched using a custom macro created by Anna Lladó and Sebastian Tosi from the Advanced Digital Microscopy Core Facility at IRB Barcelona and designed for the Fiji program. Cell counting was processed with another custom macro created by Anna Lladó. The total number of cells per × 4 field in each well is shown. A two-tail Mann–Whitney unpaired *t*-test was used to analyse statistical differences between groups.

### Invasion assay

Cells were marked with 5 μM of cell Tracker green (Invitrogen), following the manufacturer's instructions, and kept overnight in medium without FBS. Next day, 5 × 10^4^ cells were seeded in triplicates on Matrigel Invasion Chambers (BD Bioscience, cat no 354480) in medium without FBS, while the wells were loaded with complete medium. Before seeding, inserts were hydrated with 500 μl of medium for 2 h at 37 °C. Eight hours after seeding, cells were washed with PBS and fixed with 4% formalin. Cells on the apical side of each insert were scraped off, and migration to the basolateral side was visualized with an Olympus ScanR-Xcellence-TIRF widefield fluorescence microscope. Each insert was segmented in 6 × 6 squares (in total 36), and one image per square was taken. The images shown is the result of the 36 pictures stitched using a custom macro created by Anna Lladó and Sebastian Tosi from the Advanced Digital Microscopy Core Facility in IRB Barcelona and designed for the Fiji program. Cell counting was processed with another custom macro created by Anna Lladó. Total number of cells per × 4 field in each well is shown. Two tails Mann–Whitney unpaired *t*-test was used to analyse statistical differences between groups.

### Oncosphere formation assay

Cells were collected, and 1 cell per well was sorted into ultra-low attachment 96-well plates (Costar, cat no 3474). Cells were cultured in suspension in the following media: mammary epithelial basal medium (MEBM, LONZA cat no. CC-3151), supplemented with MEGM Single-Quots (containing EGF, Hydrocortisone, Insulin and GA-1000, LONZA cat no. 4136), 20 ng ml^−1^ of recombinant fibroblast growth factor (GIBCO, cat no. PHG0026) and 1X B27 without retinoic acid (GIBCO, cat no. 12787-010). Plates were placed at 37 °C, 5% CO_2_ for 15 days. Quantification was performed manually, counting total number of oncospheres under the microscope. A two-tail Mann–Whitney unpaired *t*-test was used to analyse statistical differences between groups.

### Organotypic formation assay

To form 3D organoids, cells were mixed in growth-reduced factor matrigel (BD Bioscience, cat no. 354230) in a concentration of 2,000 cells per 25 μl of matrigel. Each drop was dispensed in the center of the well from adherent 48-well plate and incubated for 15 min at room temperature. After matrigel is solidified, 250 μl of oncosphere assay media (see above) was added to each well. Every other day, media was changed, and organotypic 3D structures were grown for a total of 15 days. The total number of organoids was counted using a custom macro created by the Advance Digital Microscopy Core Facility at IRB Barcelona. The images shown are the result of the 42 pictures stitched (which recreates the whole drop) using a custom macro created by the Advanced Digital Microscopy Core Facility at IRB Barcelona and designed for the Fiji program. A two-tail Mann–Whitney unpaired *t*-test was used to analyse statistical differences between groups.

### Experimental procedures for patient data sets

We used the MSK-82 and EMC-344 data sets, which are based on HG-U133A and were combined, and the EMC-189 data set, which is based on HG-U133plus2 and was processed separately (GSE2603, GSE12276, GSE5327 and GSE2034 available at the Gene Expression Omnibus (GEO) public database). To remove systematic biases, the expression measurements were converted to *z*-scores for all genes before merging. Patient clinical records of the 615 primary tumour samples were extracted from supplemental material described by Zhang *et al.* ‘Latent bone metastasis in BCa tied to Src-dependent survival signals'^36^. Following the indications of the Cancer Cell manuscript (Supplementary Table 1, page 33 of Supplementary Material), we retrieved the metastasis site annotation from Table 8 of Bos *et al.* ‘Genes that mediate BCa metastasis to the brain^10^. The site of metastasis annotation was available for 560 out of the 615 samples. The median follow-up among all 560 patients was 4 years (range 0–14.25). The 55 patients with no time to metastasis reported were not included at any ulterior time after metastasis analysis. The median duration of follow-up was 7.667 years (range 0–14.25) for the 268 patients without metastasis and 1.917 years (range 0–9.583) for the 292 patients with metastasis.

As described elsewhere^37^, tumour specimens and mammoplasties were obtained from the Parc de Salut Mar Biobank (MARBiobanc, Barcelona, Spain), and the Valencia Clinic Hospital Biobank (Valencia, Spain), the Fundación Jiménez Díaz Biobank (Madrid, Spain). Primary breast tumours from formalin-fixed paraffin-embedded blocks were retrospectively selected from consecutive patients diagnosed between 1998 and 2000. The following criteria were required for patients enrollment: no neoadjuvant therapy; infiltrating carcinomas; operable; sufficient tissue for research purposes once fulfilled all clinical needs; and clinical follow-up. Normal breast tissue cases (*n*=20) were obtained from non-cancerous mammoplasties.

Tumour–node–metastasis was categorized using the staging system of the American Joint Committee on Cancer (AJCC). ER and PR were assessed by immunohistochemistry (IHC) (SP1 and PgR636 clones, respectively; Dako, Carpinteria, CA) establishing positivity criteria in ≥1% of nuclear tumour staining. Histological grade was defined following Scarff–Bloom–Richardson modified by Elston and Ellis, Histopathology (1991) 19: 403-410. FISH assays were used for HER2 amplification (PathVysion; Abbott Laboratories, Abbott Park, IL). Ki-67 was analysed by IHC (MIB1 clone; Dako). *BRCA1* and *BRCA2* gene status was tested in patients that required genetic counseling. The Ethics Committees of the three hospitals approved the study and informed consent from all subjects was secured.

## Additional information

**Accession codes:** The gene expression data used in Fig. 2b have been deposited in the Gene Expression Omnibus (GEO) public functional genomics data repository under the accession code GSE61164.

**How to cite this article:** Slebe, F. *et al.* FoxA and LIPG endothelial lipase control the uptake of extracellular lipids for breast cancer growth. *Nat. Commun.* 7:11199 doi: 10.1038/ncomms11199 (2016).

## Supplementary Material

Supplementary Information Supplementary Figures 1-6, Supplementary Tables 1-3, Supplementary Methods and Supplementary References.
