# Supplementary material for: FoxA and LIPG endothelial lipase control the uptake of extracellular lipids for breast cancer growth
Source: Nat Commun. 2016 Apr 5;7:11199. doi: 10.1038/ncomms11199 (PMC4822041; doi:10.1038/ncomms11199)
Supplement: Supplementary Information — Supplementary Figures 1-6, Supplementary Tables 1-3, Supplementary Methods and Supplementary References. [file ncomms11199-s1.pdf]

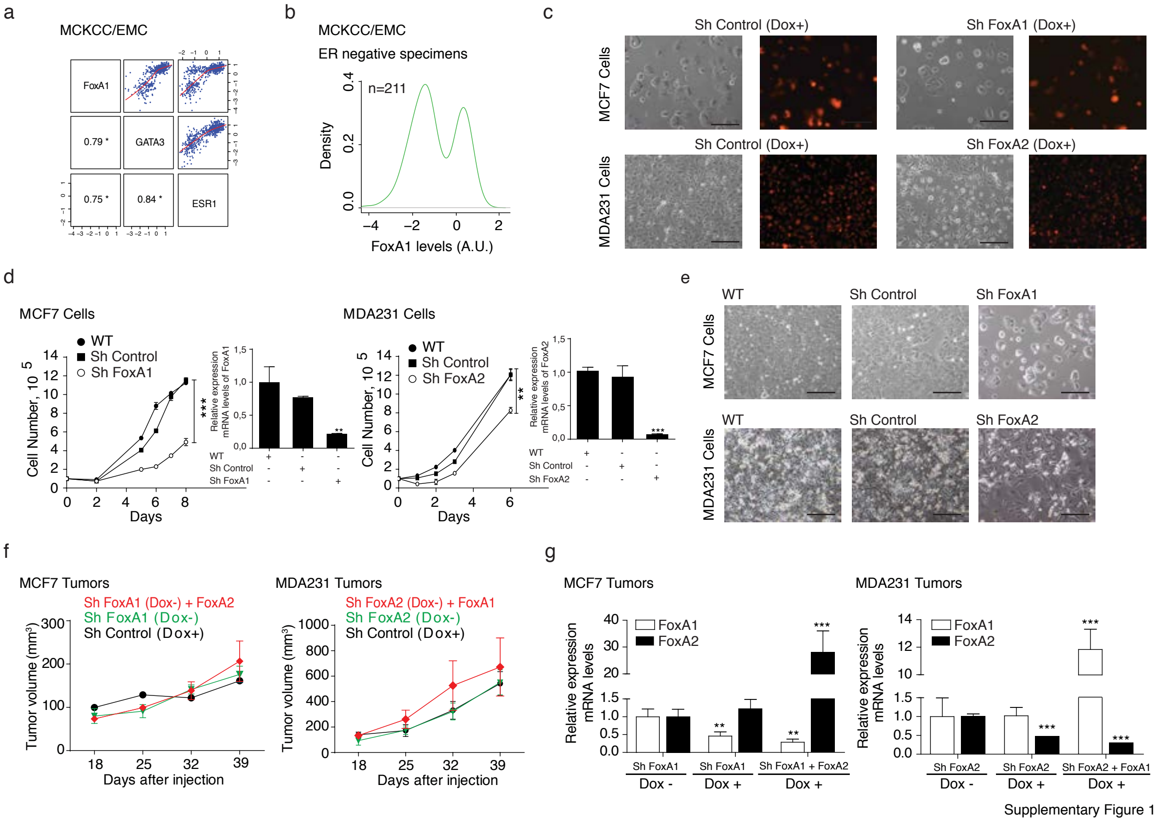

Figure S1 related to Figure 1.- FoxA family of transcription factors and breast cancer growth.

(**a**) Correlation plots between the expression of the indicated genes in human breast cancer specimens (MCKC/EMC set). The values are the Spearman estimates of the correlations, with the asterisk showing that the significance p values are <0.05.

(**b**) Kernel density plot of FoxA1 mRNA expression in human breast cancer specimens (MCKC/EMC set). Plot represents specimens without estrogen receptor expression (ER−).

(**c**) tRFP detection in MCF7, MDA231 and their derivative cells. Cell populations were cultured in the presence of doxycycline for 6 days to induce the expression of tRFP concomitantly with the expression of FoxA short hairpin.

(**d**) FoxA1 and FoxA2 gene expression analysis by qRT-PCR and *in-vitro* growth of shFoxA1 MCF7 cells and shFoxA2 MDA231 cells compared with shControl cells. P value is the result of T-test. Data are average ± SEM; n=3. ** $p \leq 0.01$, *** $p \leq 0.001$.

(**e**) Light microscopy of shFoxA1 MCF7 cells and shFoxA2 MDA231 cells compared with shControl cells. Pictures were taken at the last point of the growth curve from figure S1D.

(**f**) Tumor growth of control cell populations inoculated in Balb/c nude mice are determined at the indicated time points. Black lines: MCF7 and MDA231 sh control tumors treated with doxycycline, green lines: MCF7 sh FoxA1 or MDA231 sh FoxA2 tumors treated without doxycycline, red lines: MCF7 sh FoxA1 or MDA231 sh FoxA2 tumors overexpressing FoxA2 or FoxA1 respectively without doxycycline treatment. P value is the result of T-test. Data are average ± SEM; n= 3–5 tumors.

(**g**) FoxA1 (white bars) and FoxA2 (black bars) mRNA levels of the indicating MCF7 and MDA231 tumor xenografts analyzed by qRT-PCR. P value is the result of T-test. Data are average ± SEM; n= 5–8 tumors. ** $p \leq 0.01$, *** $p \leq 0.001$.

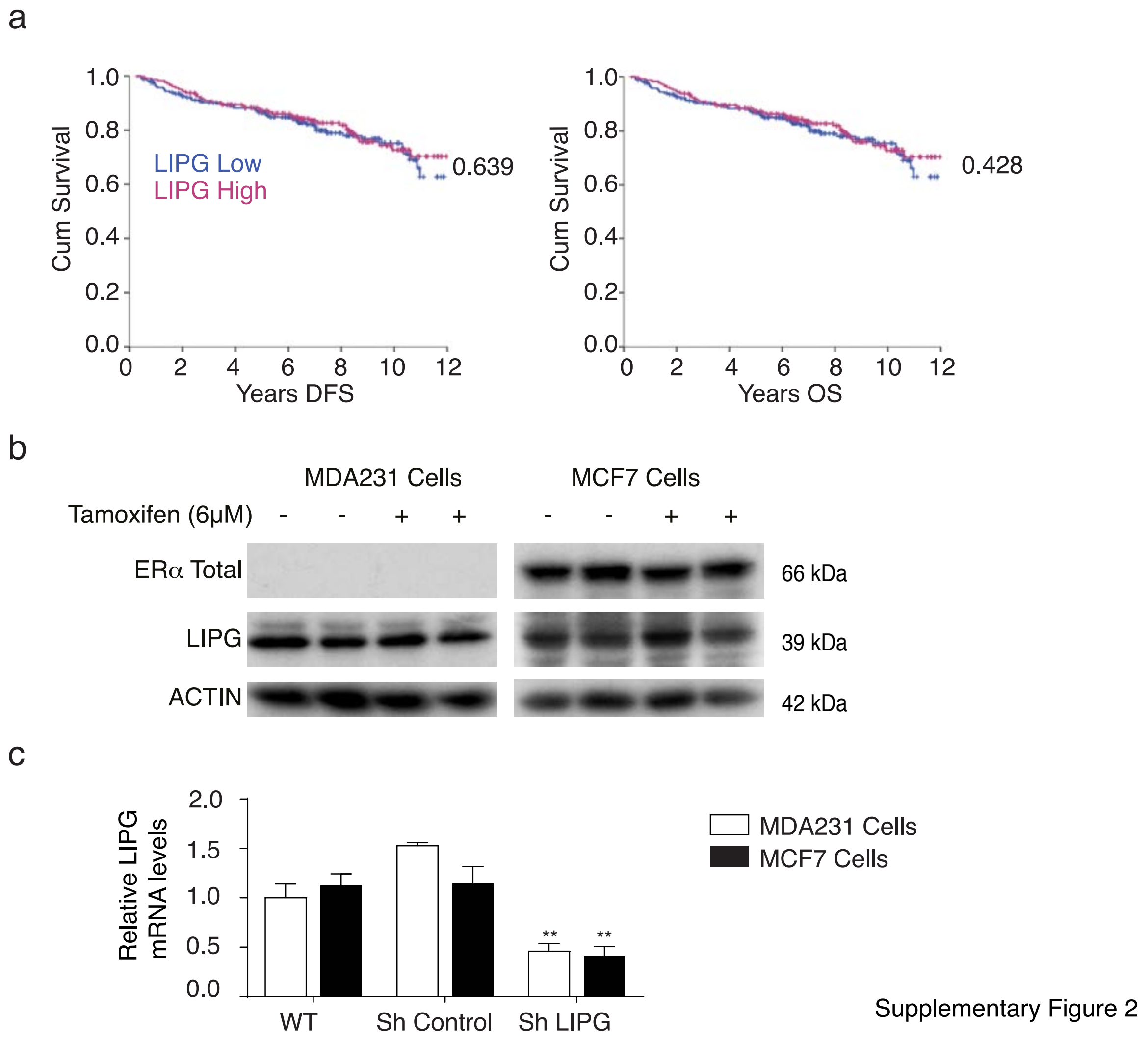

Supplementary Figure 2.- Clinical outcome for LIPG expression in Spanish data set and LIPG expression in tamoxifen treated cells or LIPG depleted cells.

(**a**) LIPG association with disease-free survival and overall survival in breast cancer specimens (Spanish set). Low (blue lines) and high (red lines) represent LIPG protein expression levels

(**b**) LIPG and total ERα immunoblots of MCF7 and MDA231 cells treated with or without tamoxifen (6μM). The blot shown is representative of 3 independent experiments.

(**c**) LIPG gene expression analysis by qRT-PCR in LIPG depleted MCF7 and MDA231 cells compared with shControl cells. P value is the result of T-test. Data are average ± SEM; n= 3. ** $p \leq 0.01$.

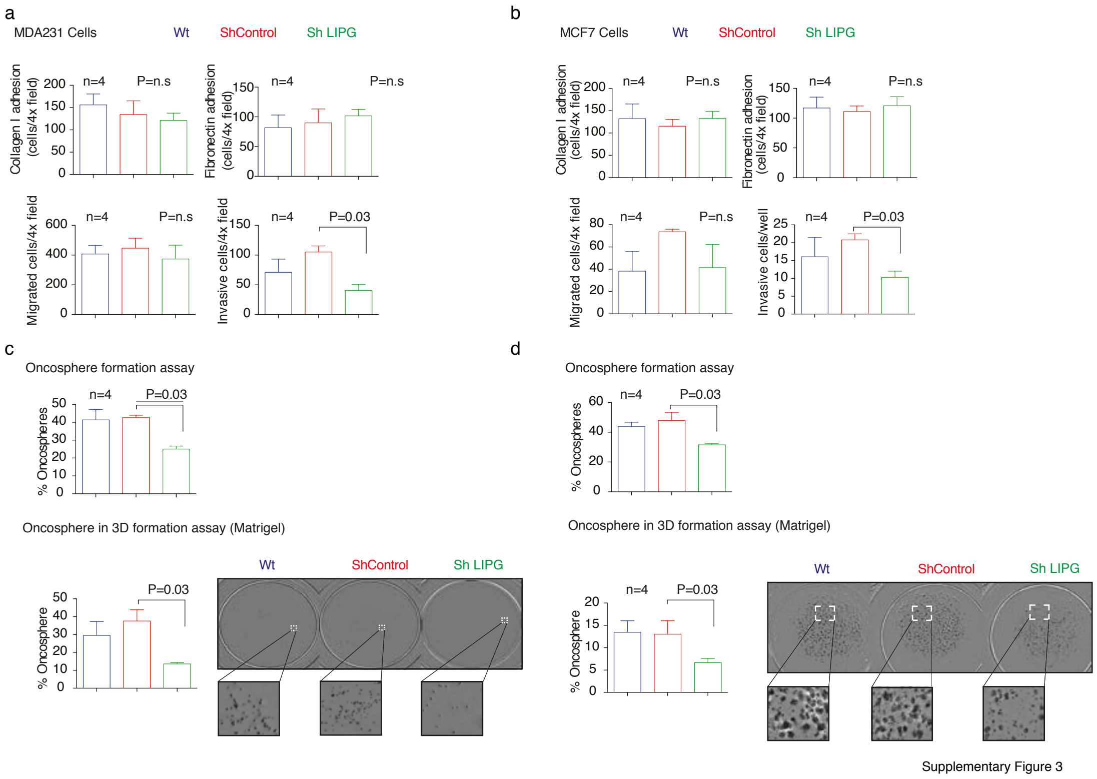

Supplementary Figure 3.- LIPG drives invasion and self-renewal properties.

(**a and b**) Migration, adhesion and invasion assays in MCF7 and MDA231 LIPG depleted cells compared with ShControl cells . P value is the result of T-test. Data are average ± SEM; n= 4.

(**c and d**) Microscopy images and quantification of oncospheres in LIPG depleted MCF7 and MDA231 cells compared with ShControl cells. P value is the result of T-test. Data are average ± SEM; n= 4.

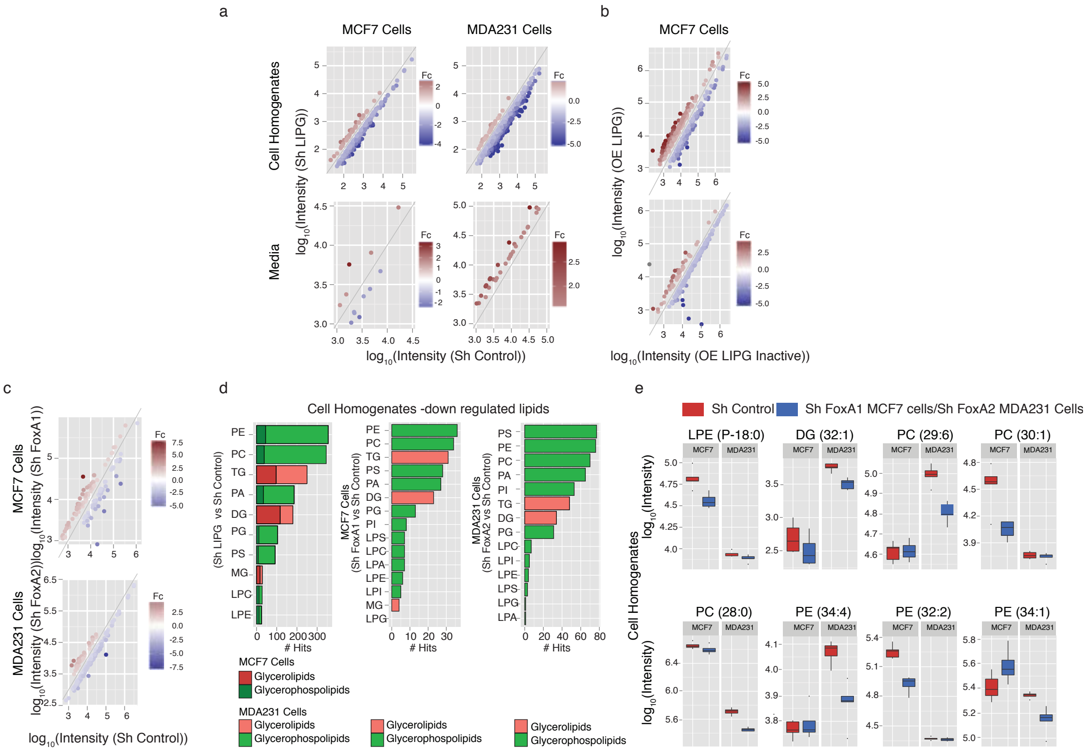

Supplementary Figure 4

Supplementary Figure 4.- Lipidomic approach to determine the metabolic reprograming in LIPG, FoxA1 or FoxA2-depelteted breast cancer cells.

(**a**) Down-regulated (blue dots) and up-regulated (red dots) metabolic features found in the cell homogenates or media of MCF7 or MDA231 shLIPG cells compared to shControl cells. The feature intensity is a $\log_{10}$ representation of m/z peak intensities by mass spectrometry.

(**b**) Down-regulated (blue dots) and up-regulated (red dots) metabolic features found in the cell homogenates or media of MCF7 cells overexpressing WT LIPG compared to MCF7 cells overexpressing inactive LIPG. The feature intensity is a $\log_{10}$ representation of m/z peak intensities by mass spectrometry.

(**c**) Down-regulated (blue dots) and up-regulated (red dots) metabolic features found in the cell homogenates of shFoxA1 MCF7 or shFoxA2 MDA231 cells compared to shControl cells. The feature intensity is a $\log_{10}$ representation of m/z peak intensities by mass spectrometry.

(**d**) Down-regulated glycerolipids, glycerophospholipids, and sphingolipids in cell homogenates of LIPG, FoxA1 or FoxA2 -depleted versus shControl cells.

(**e**) Certain down-regulated lipid species in shLIPG MCF7/MDA231 cells (previously identified in Figure 4b) are also decreased in FoxA1-depleted MCF7 and FoxA2-depleted MDA231 cells. Lipid species intensities are represented in box plots as $\log_{10}$ intensity. ShControl cells (red box), and shLIPG cells (blue box). P-values are <0.05 and calculated using Welch's t-test, n=5.

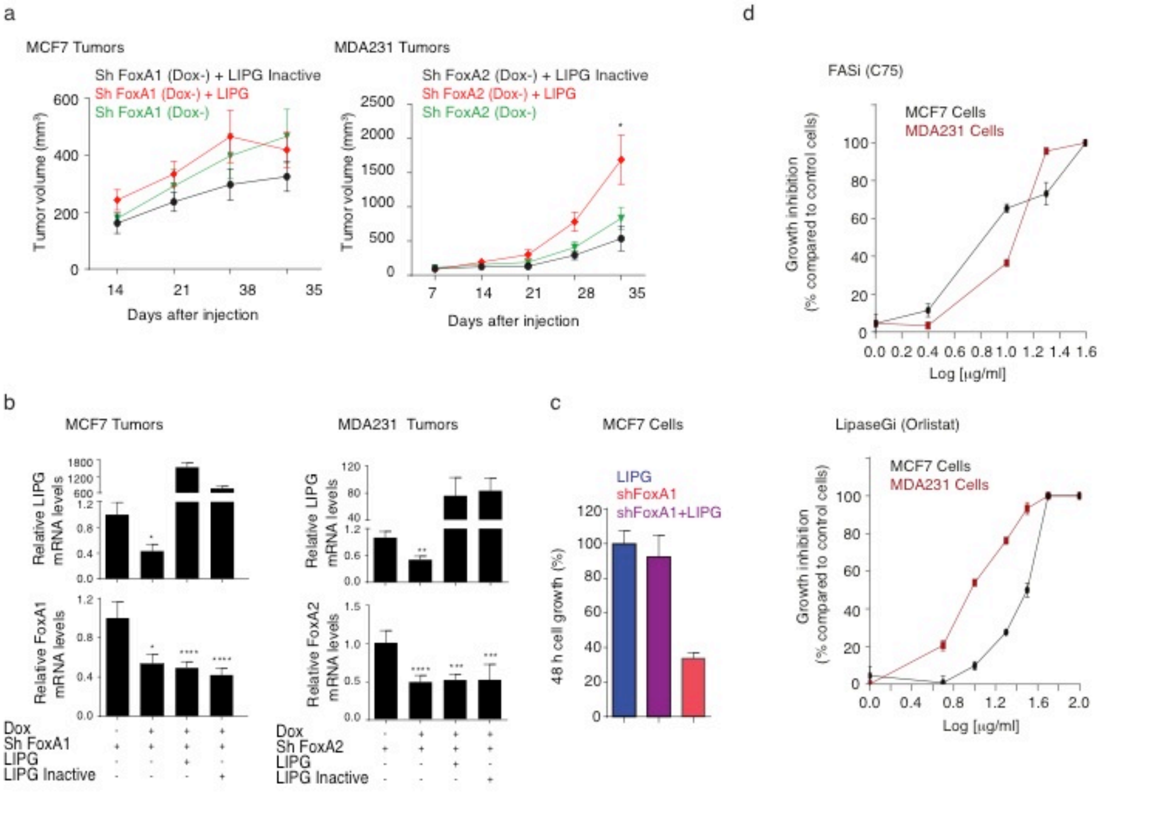

Supplementary Figure 5

Supplementary Figure 5.- LIPG activity is essential for breast tumor growth.

(**a**) Tumor growth of control cell populations inoculated in Balb/c nude mice are determined at the indicated time points. Green lines: MCF7 sh FoxA1 or MDA231 sh FoxA2 tumors without doxycycline treatment, red lines: MCF7 sh FoxA1 or MDA231 sh FoxA2 tumors overexpressing WT LIPG without doxycycline treatment, black lines: MCF7 sh FoxA1 or MDA231 sh FoxA2 tumors overexpressing inactive LIPG without doxycycline treatment. P value is the result of T-test. Data are average ± SEM; n= 3–5 tumors.

(**b**) FoxA1, FoxA2 and LIPG gene expression analysis by qRT-PCR in the corresponding tumor population *ex-vivo*. P value is the result of T-test. Data are average ± SEM; n= 5–8 tumors. * p≤0.05, ** p≤0.01, *** p≤0.001, **** p≤0.0001.

(**c**) Cell growth inhibition (%) of MDA231 and MCF7 treated for 48 h with FAS inhibitor (C75) and/or Lipase inhibitor (Orlistat) and compared with DMSO treatment (control). Concentration of drugs are represented as log.

(**d**) Cell Growth (%) of FoxA1 depleted MCF7 cells with or without WT LIPG expression for 48h. P value is the result of T-test. Data are average ± SEM; n=3

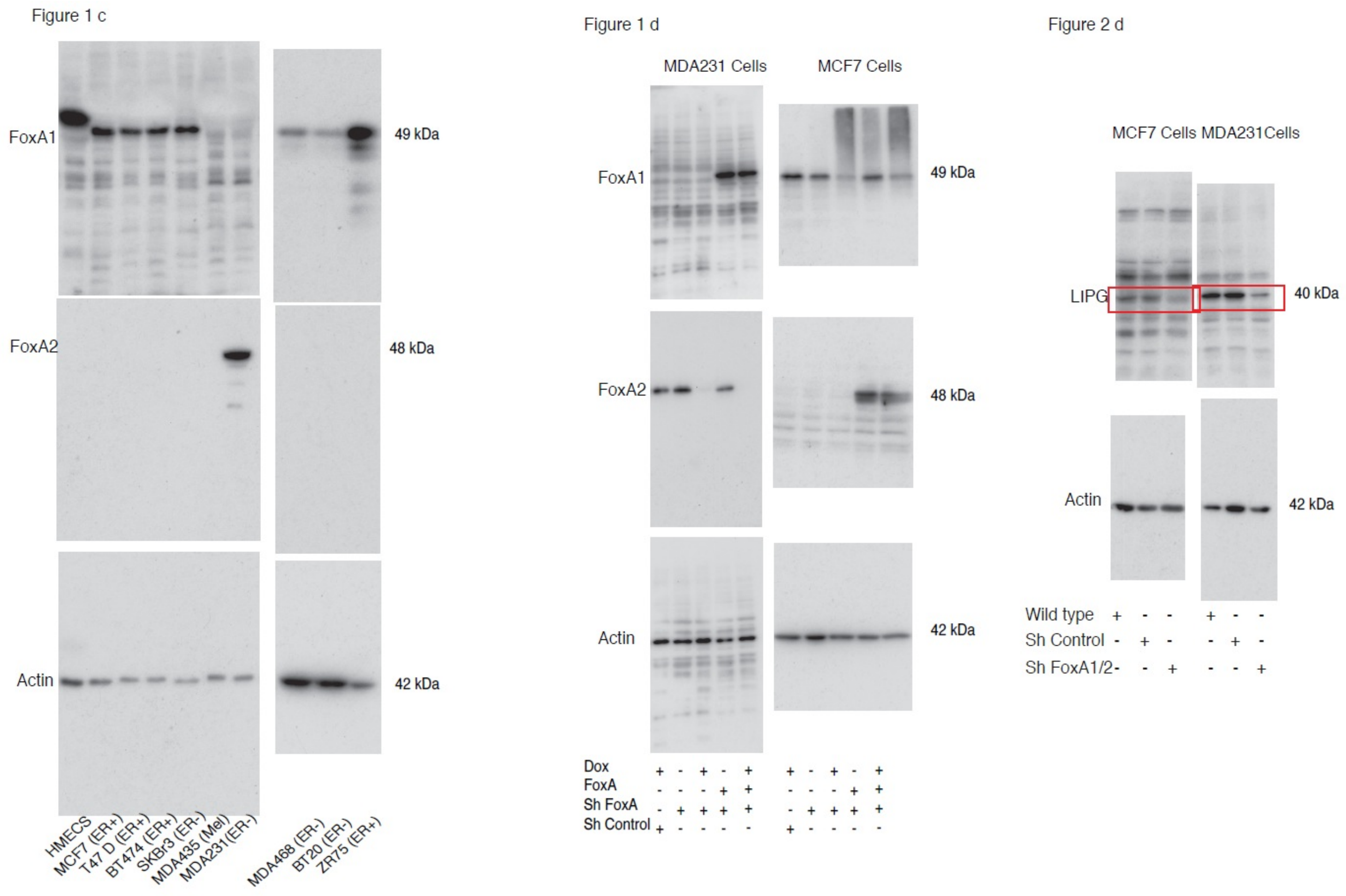

Supplementary Figure 6

Supplementary Figure 6.-Complete western blots of the gels depicted in the manuscript

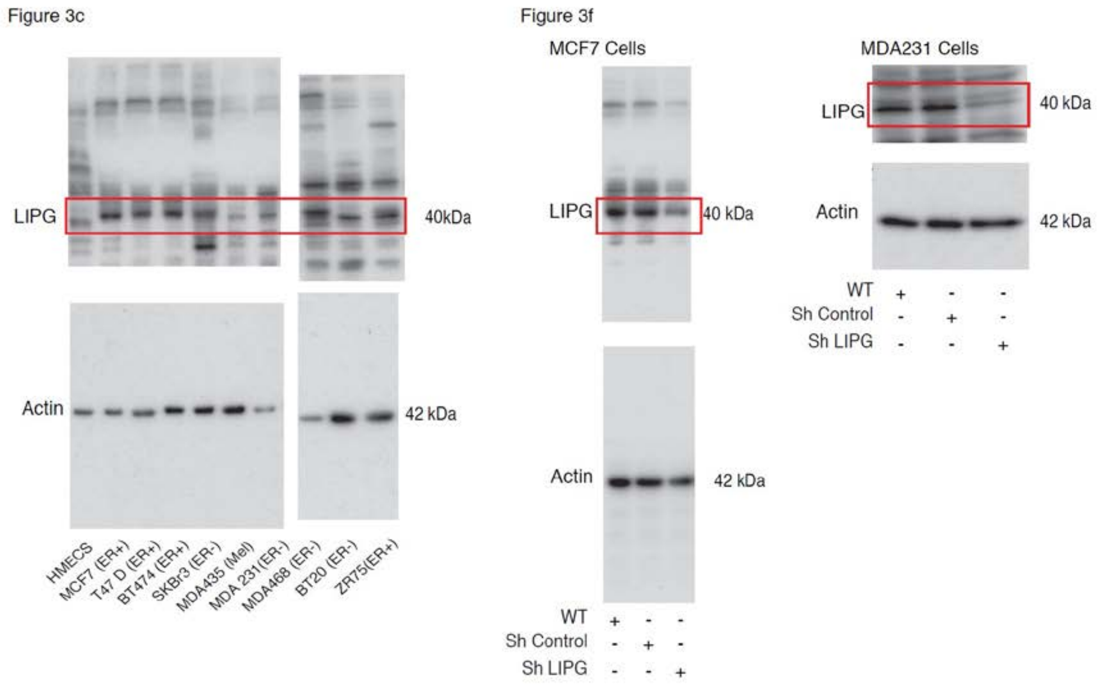

Supplementary Figure 6.- (Continuation) Complete western blots of the gels depicted in the manuscript

Figure 5b

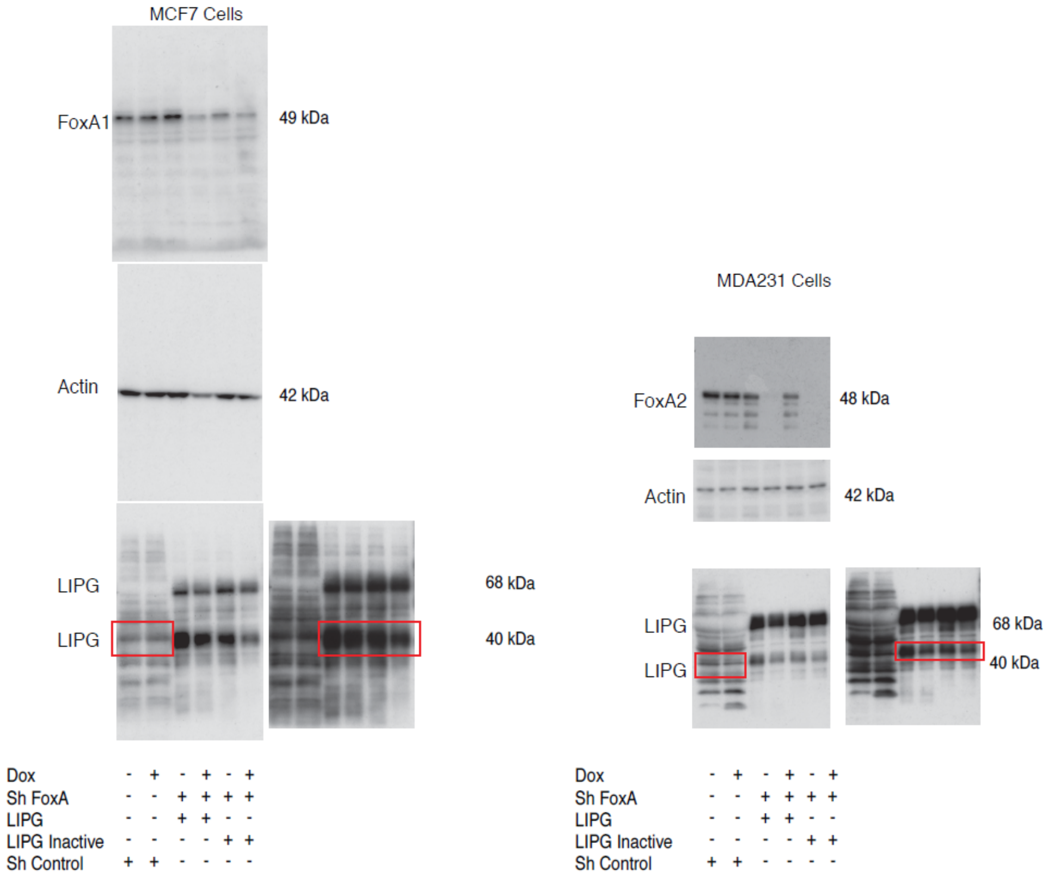

Suplementary Figure 2b

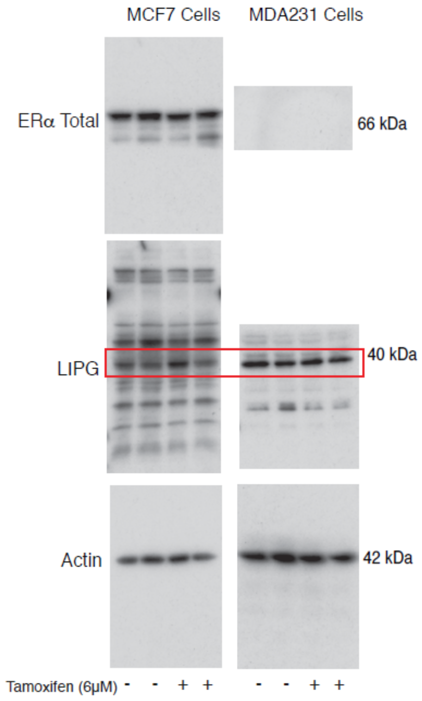

Supplementary Figure 6.- (Continuation) Complete western blots of the gels depicted in the manuscript

Supplementary Table 1.- FoxA1 and FoxA2 expression in Spanish data set

	Number of Tumors	Percentage of Tumors
Total Tumors	439	100.0%
Luminal-like (ER+)	326	74.3%
FoxA1+	289	65.8%
FoxA2+	0	0.0%
Non-luminal-like (ER−)	113	25.7%
FoxA1+	62	14.1%
FoxA2+	6	1.4%
FoxA+ Tumors	357	81.3%

Supplementary Table 2.- CDH11, Bcl2 and LIPG expression in the Affymetrix analysis

MCF7 Cells			
**(Sh FoxA1 (Dox-) vs. ShFoxA1 (Dox+))**		**Sh FoxA1 (Dox+) vs. Sh FoxA1(Dox+)+FoxA2**	
Symbol	Fold Change	Symbol	Fold Change
CDH11	-3.71	CDH11	2.55
Bcl2	3.06	Bcl2	-3.37
LIPG	3.23	LIPG	-5.94

MDA231 Cells			
**Sh FoxA2 (Dox-) vs. ShFoxA2 (Dox+)**		**Sh FoxA2 (Dox+) vs. Sh FoxA2(Dox+)+FoxA1**	
Symbol	Fold Change	Symbol	Fold Change
CDH11	-5.18	CDH11	3.74
Bcl2	4.98	Bcl2	-3.29
LIPG	5.19	LIPG	-3.93

Supplementary Table 3.- LIPG expression in Spanish data set

	Number of Tumors	Percentage of Tumors
Total Tumors	439	100.0%
Luminal-like (ER+)	326	74.3%
LIPG+	279	63.6%
ER−Non-Luminal like (ER-)	113	25.7%
LIPG+	89	20.3%
LIPG+ Tumors	368	83.8%

Supplementary Methods

FACS sorting

Tumors were isolated as described previously [1]. Subcutaneous tumors were removed from the mice, and a fraction of the tumor was cut into pieces and incubated in buffer containing Collagenase IV (Sigma Aldrich) for 30 min at 37ºC. Samples were further homogenized using an 18G needle and then passed through a 70-µm mesh filter and centrifuged at 1200 rpm for 5 min at 4ºC. The pellet obtained was incubated in an ammonium chloride buffer for 3 min at room temperature to lyse erythrocytes. It was then washed, resuspended in DMEM 10% FBS containing 10 µg/mL PI, and sorted in a FACS Aria 2.0 (BD). Cells were sorted on the basis of GFP and RFP expression and then collected in DMEM 10% FBS. After collection, total RNA was extracted.

Animal studies and xenograft models

All animal work was done in accordance with the protocol approved by the institutional Animal Care and Use Committee of the Institute for Research in Biomedicine (IRB Barcelona) and the Parc Científic de Barcelona (CEEA). For assays involving subcutaneous implanted tumors, cells were harvested by trypsinization, washed twice with PBS, and counted. They were then resuspended in 50 µl growth factor-reduced Matrigel. Female Balb/c nude mice 4-6 weeks old were treated with or without doxycycline in drinking water throughout the experiment. After the second week of treatment, mice were anesthetized using a cocktail of 100 mg/kg ketamine and 10 mg/kg xylazine. A cell suspension of $1x10^6$ cells was inoculated subcutaneously. Tumor growth was monitored weekly. An estrogen pellet was implanted in mice injected with MCF7 cells (ER+). Tumor growth rates were analyzed by measuring tumor length (L) and width (W), and

calculating tumor volume based on the formula *Volume* = $\pi L W^2/6$. Mice were killed at day 35 or 39 post cell inoculation, and tumors were extracted. A fraction of the tumor was used to purify tumor cells. Another fraction was directly frozen in dry ice for protein and RNA extraction. An assessment of the number of animals required for each procedure has been performed using Statistical Power Analysis and taking into consideration the above statistical test, significance level of 5% and statistical power of 80%. An estimate of variance has been inferred from previous experiments, specially considering the variability of metastatic burdens in xenograft assays.

Plasma cholesterol measurement

Plasma was obtained from the extracted blood by centrifugation (1650 *g*, 20 min) at 4°C and stored at -20°C until assayed. Plasma total cholesterol was determined spectrophotometrically using a commercial kit (HORIBA ABX, Montpellier, France).

Metabolomic studies

Lipid extraction method

Fresh media (media without cells) and culture media removed from cells were freeze in nitrogen liquid and lyophilized. The culture plates with the remaining cells were freeze in nitrogen liquid and then placed on dry ice. Cells were scrapped immediately in 1 mL of cold ultrapure water, after which the solution was frozen and lyophilized. Lipids were extracted from lyophilized samples (cell homogenates and media) by adding 570 μL of a cold mixture of dichloromethane/methanol (2:1 v/v). The resulting suspension was vortexed and bath-sonicated for 5 min. We subsequently added 120 μL of cold water and vortexed samples again. Organic and aqueous layers were allowed to equilibrate for 10 min at room temperature. Cell lysates were centrifuged (15,000 rpm, 15 min at 4°C), and the organic phase (lipidic) was collected for drying under a stream of nitrogen. Lipid pellets were resuspended in 300 μL of

acetonitrile/isopropanol/water (65:30:5 v/v) for LC-MS analysis. 100 μL of culture media was lyophilized and subsequently resuspended in dichloromethane/methanol (2:1 v/v) following the same procedure as that used for cells, with the exception that lipid pellets were resuspended in 200 μL (acetonitrile: isopropanol: water (65:30:5 v/v).

LC/MS analysis

Untargeted LC/MS analyses were performed using an UHPLC system (1200 series, Agilent Technologies) coupled to a 6550 ESI-QTOF MS (Agilent Technologies) operating in positive (ESI+) or negative (ESI–) electrospray ionization mode. Lipids were separated by reverse phase chromatography with an Acquity UPLC C8 column (150 x 2.1 mm, 1.8 μm). Mobile phase A = water/acetonitrile (60:40) (10 mM ammonium formate and 0.1% formic acid) and B = isopropanol/acetonitrile (95:5) (10 mM ammonium formate, 0.1% formic acid and 0.1% H2O). Solvent modifiers, such as 0.1% formic acid and 10 mM ammonium formate, were used to enhance ionization, as well as to improve the LC resolution in both positive and negative ionization modes. The elution gradient started at 32% B (time 0–1 min), increased to 60% of B (time 1–4 min) and increased again to 100% B over 11 min (time 4–15 min). The injection volume was 2 μL for cell lipids and 5 μL for media lipids. ESI conditions: gas temperature, 150 °C; drying gas, 13 L min–1; nebulizer, 35 psig; fragmentor, 150 V; and skimmer, 65 V. The instrument was set to work over the m/z range 50–1200 with an acquisition rate of 3 spectra/sec. For compound identification, MS/MS analyses were performed in targeted mode, and the instrument was set to acquire spectra over the m/z range 50–1000, with a default iso width (the width at half-maximum of the quadrupole mass bandpass used during MS/MS precursor isolation) of 4 m/z. The collision energy was fixed at 20 V.

Data analysis

Data from cells and media were treated in the same way. LC/MS (ESI+ and ESI− mode) data were processed using the XCMS [2] software (version 1.34.0) to detect and align mzRT features. A feature is defined as a molecular entity with a unique m/z and a specific retention time. XCMS analysis of these data provided a matrix containing the retention time, m/z value, and integrated peak area of each feature for each sample of cells and culture medium. We constrained the initial number of features by means of the following criteria: only features above an intensity threshold of 5,000 and 2,000 counts for the analysis of cells and media respectively were retained for further statistical analysis. Quality control samples (QCs) consisting of pooled cells and media samples from each condition were injected at the beginning and periodically every four samples. The performance of the LC/MS platform for each mzRT feature detected in MDA-231 and MCF7 samples was assessed by calculating the relative standard deviation of these features on pooled samples (CV_{QC}), following [3]. We normalized peak intensities to dry weight of cells to account for cell number differences between plates. Next, the intensities of the mzRT features in sh LIPG and sh control samples were compared using Welch's t-test in both MCF7 and MDA-231 cell lines and in media. Differentially regulated lipids (p value<0.05 and fold>1.5) were retained for further tandem MS characterization. Lipid structures were identified by matching tandem MS spectra against reference standards in LIPIDMAPS [4] and/or LipidBlast [5] databases.

Protein Structure Modeling

The structure of the N-terminal domain of human endothelial lipase precursor (residues 47 to 346 in uniprot Q9Y5X9) was modeled with the web tools provided by SWISS-MODEL (swissmodel.expasy.org) by using the structure of pancreatic lipase-related protein 1 as a template (pdb code 2ppl), with which it shares 38% sequence identity. With the exception of the lid region and the loops connecting secondary structure elements, which are expected to present substantial dynamics, the quality of the modeled structure was considered good by the QMEAN server (swissmodel.expasy.org/qmean/cgi/index.cgi).

Supplementary References

1. Merlos-Suarez A*, et al.* The intestinal stem cell signature identifies colorectal cancer stem cells and predicts disease relapse. *Cell stem cell* **8**, 511-524 (2011).

2. Smith CA, Want EJ, O'Maille G, Abagyan R, Siuzdak G. XCMS: processing mass spectrometry data for metabolite profiling using nonlinear peak alignment, matching, and identification. *Analytical chemistry* **78**, 779-787 (2006).

3. Vinaixa M, Samino S, Saez I, Duran J, Guinovart JJ, Yanes O. A Guideline to Univariate Statistical Analysis for LC/MS-Based Untargeted Metabolomics-Derived Data. *Metabolites* **2**, 775-795 (2012).

4. Fahy E, Sud M, Cotter D, Subramaniam S. LIPID MAPS online tools for lipid research. *Nucleic acids research* **35**, W606-612 (2007).

5. Kind T, Liu KH, Lee do Y, DeFelice B, Meissen JK, Fiehn O. LipidBlast in silico tandem mass spectrometry database for lipid identification. *Nature methods* **10**, 755-758 (2013).
